# Competition and succession among coral endosymbionts

**DOI:** 10.1002/ece3.5749

**Published:** 2019-10-30

**Authors:** Shelby E. McIlroy, Ross Cunning, Andrew C. Baker, Mary Alice Coffroth

**Affiliations:** ^1^ Graduate Program in Evolution, Ecology and Behavior State University of New York University at Buffalo Buffalo New York; ^2^ Swire Institute of Marine Science School of Biological Science The University of Hong Kong Hong Kong; ^3^ Department of Marine Biology and Ecology Rosenstiel School of Marine and Atmospheric Science University of Miami Miami Florida; ^4^ Department of Geology State University of New York University at Buffalo Buffalo New York; ^5^ Present address: Swire Institute of Marine Science School of Biological Science The University of Hong Kong Hong Kong; ^6^ Present address: Daniel P. Haerther Center for Conservation and Research John G. Shedd Aquarium Chicago Illinois

**Keywords:** competition, microbiome, succession, Symbiodineaceae, symbiosis

## Abstract

Host species often support a genetically diverse guild of symbionts, the identity and performance of which can determine holobiont fitness under particular environmental conditions. These symbiont communities are structured by a complex set of potential interactions, both positive and negative, between the host and symbionts and among symbionts. In reef‐building corals, stable associations with specific symbiont species are common, and we hypothesize that this is partly due to ecological mechanisms, such as succession and competition, which drive patterns of symbiont winnowing in the initial colonization of new generations of coral recruits. We tested this hypothesis using the experimental framework of the de Wit replacement series and found that competitive interactions occurred among symbionts which were characterized by unique ecological strategies. Aposymbiotic octocoral recruits within high‐ and low‐light environments were inoculated with one of three Symbiodiniaceae species as monocultures or with cross‐paired mixtures, and we tracked symbiont uptake using quantitative genetic assays. Priority effects, in which early colonizers excluded competitive dominants, were evidenced under low light, but these early opportunistic species were later succeeded by competitive dominants. Under high light, a more consistent competitive hierarchy was established in which competitive dominants outgrew and limited the abundance of others. These findings provide insight into mechanisms of microbial community organization and symbiosis breakdown and recovery. Furthermore, transitions in competitive outcomes across spatial and temporal environmental variation may improve lifetime host fitness.

## Graphical abstract

Reef‐building corals rely on a genetically diverse group of dinoflagellate symbionts to meet their nutritional needs. The ecological mechanisms that regulate symbiont communities are not well understood. Here, we show that competition and succession among symbiont species are important in the early stages of the symbiosis.

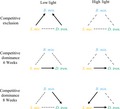

## 1 INTRODUCTION

Many biological communities are structured by obligate, mutualistic symbioses consisting of a relatively long‐lived macro‐organism that provides habitat for diverse short‐lived microbial symbionts. Genetic inquiries into the symbiont communities of yeast‐termite, fig tree‐fig wasp, plant‐fungi, and coral‐dinoflagellate symbioses have revealed the presence of dozens of symbiont species, whose influence on host fitness can range from mutualistic to parasitic across space and time (Baker, Freeman, Wong, Fogel, & Knowlton, 2018; Heath, Burke, & Stinchcombe, 2012; Lesser, Stat, & Gates, 2013; Livne‐Luzon et al., 2017; Prillinger et al., 1996). Symbiont genetic diversity may be beneficial if symbiont types provide distinct and/or complementary resources to their host (sensu Palmer et al., 2010; Stachowicz & Whitlatch, 2005; Wagg, Jansa, Stadler, Schmid, & Heijden, 2011), especially if these resources vary by environment. However, competitive interactions among diverse symbionts for access to host‐derived resources may also destabilize the symbiosis in the short term (Cushman & Addicott, 1989; Frank, 1996) or result in ecologically suboptimal holobionts in the long term (Afkhami, Rudgers, & Stachowicz, 2014; Miller, 2007; Palmer, Young, & Stanton, 2002). Thus, holobionts that can maintain diversity/flexibility in symbiotic associations in either space or time while minimizing antagonism and parasitism may achieve a wider and more dynamic niche space (Jandér & Steidinger, 2017; Livne‐Luzon et al., 2017; Palmer, 2003; Palmer et al., 2010; Stachowicz & Whitlatch, 2005).

Within multispecies symbioses, dynamic networks of direct and indirect effects, well beyond traditional pairwise interactions (Stanton, 2003), determine symbiont community structure via interactions among multiple co‐occurring symbionts, their host, and the environment (Palmer, Stanton, & Young, 2003). Environmental conditions may alter competitive hierarchies among symbionts as different resources become more or less limiting, and both host and symbionts may modify the *in‐hospite* environment in ways that can either facilitate or exclude additional symbiont types (Palmer et al., 2002; Wangpraseurt, Larkum, Ralph, & Kühl, 2012). During initial symbiont uptake in horizontally transmitting hosts (e.g., the majority of corals), priority effects, in which early arrivals are less prone to displacement, can change the trajectory of symbiont community succession and/or allow for species coexistence (Fukami, 2015; Fukami et al., 2010; Halliday, Umbanhowar, & Mitchell, 2017; Palmer et al., 2002).

Despite the role of the coral‐dinoflagellate symbiosis in supporting the most diverse marine ecosystems in the world, the ecological mechanisms that structure *in‐hospite* Symbiodiniaceae communities are poorly understood. In adult corals, a single, predictable symbiont species generally dominates the *in‐hospite* symbiont community (Goulet, 2006; Parkinson & Baums, 2014). However, the majority of coral juveniles take up symbionts from the environment anew each generation, initially establishing symbiosis with a genetically diverse subset of locally available Symbiodiniaceae, including types not typically found in adults (Coffroth, Lewis, Santos, & Weaver, 2006; Coffroth, Santos, & Goulet, 2001; Gómez‐Cabrera, Ortiz, Loh, Ward, & Hoegh‐Guldberg, 2008; Little, Oppen, & Willis, 2004; Poland et al., 2013; Reich, Robertson, & Goodbody‐Gringley, 2017; Yamashita, Suzuki, Hayashibara, & Koike, 2013). Winnowing and/or restructuring of symbiont communities (i.e., symbiont switching/shuffling; Baker, 2003) can occur during host ontogeny (Abrego, Oppen, & Willis, 2009; McIlroy & Coffroth, 2017; Poland & Coffroth, 2017; Poland et al., 2013), in response to environmental heterogeneity (Chen, Wang, Fang, & Yang, 2005; Rowan, 2004; Rowan & Knowlton, 1995), or through stress‐induced loss and subsequent re‐establishment of symbiont communities (i.e., coral “bleaching” and recovery; Baker, 2001; Baker, Starger, McClanahan, & Glynn, 2004; Cunning, Silverstein, & Baker, 2015; Jones, Berkelmans, Oppen, Mieog, & Sinclair, 2008; Rowan, Knowlton, Baker, & Jara, 1997; Toller, Rowan, & Knowlton, 2001).

**Figure 1:**
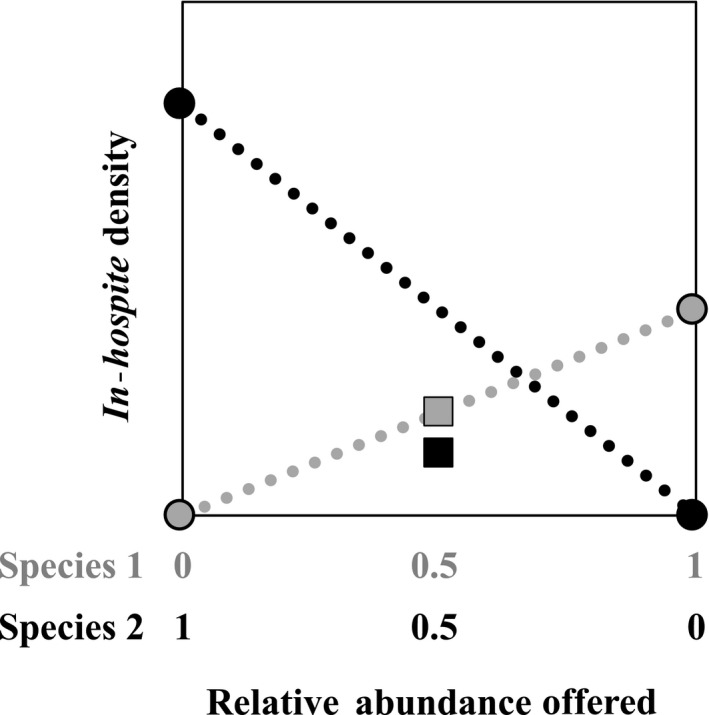
de Wit replacement series model for symbiont competition. Two potential symbiont species are offered at ratios of 1:0, 0.5:0.5, or 0:1. *In‐hospite* densities of each symbiont species measured within monocultures (circles) are used to model expected *in‐hospite* densities in the absence of competition (dashed lines). Within duoculture, measured *in‐hospite* densities that fall near (gray square) or significantly below (black square) expected values signify the absence or presence of competition, respectively

In this study, we focused on the role of competition and succession in the initial establishment of symbiont communities in newly settled coral recruits. We adapted the framework and basic expectations of the de Wit replacement series design (De Wit, 1960; Harper, 1967) (Figure 1) to evaluate competition, coexistence, and species turnover within newly available host habitat. We offered three Symbiodiniaceae species as monocultures and as three cross‐paired mixtures (0.5:0.5 ratio) to aposymbiotic octocoral recruits (*Briareum asbestinum*) and used quantitative genetic assays to determine the presence and abundance of each species within each coral recruit. We considered three models of competitive opportunistic niche exploitation: (a) competitive exclusion (one symbiont excludes another from entering into symbiosis at detectable levels) which would favor the first symbiont to enter symbiosis; (b) competitive dominance (in which one symbiont reduces the abundance of a co‐occurring symbiont) which would favor fast proliferation; and (c) a null model (no competition), in which symbiont uptake would follow the availability of each type in the environment regardless of whether additional symbiont types were present (Figure 1). We also tested whether these interactions are modulated by light levels as this is a known environmental factor that influences Symbiodiniaceae distributions in nature (Kemp, Fitt, & Schmidt, 2008; Rowan et al., 1997).

## 2 METHODS

**Figure 2:**
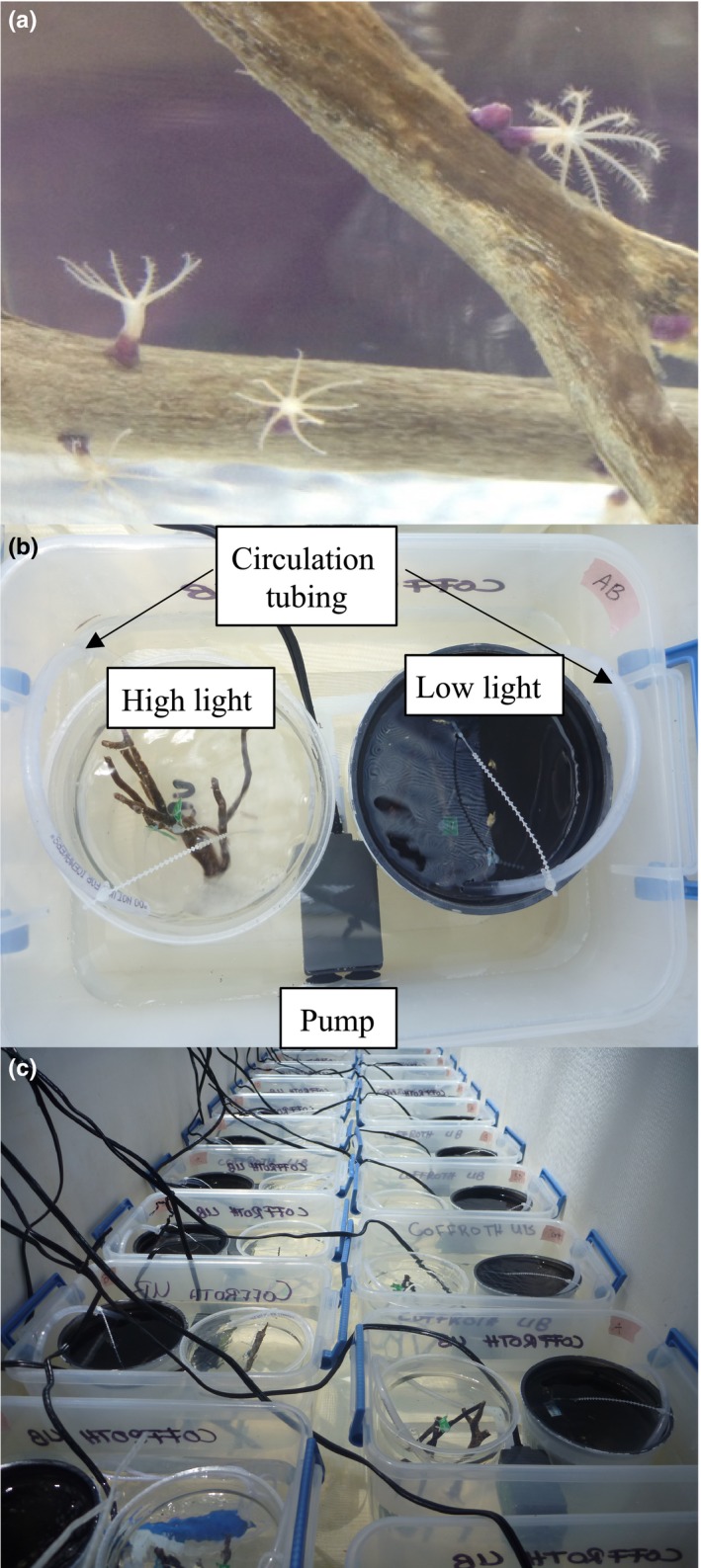
Experimental set up. (a) Aposymbiotic, single polyp recruits of *Briareum asbestinum* were settled onto dead gorgonian branches. (b) Those branches were distributed among the high‐light and low‐light treatments within a symbiont treatment tank. Cultured *Symbiodinium* (A only, B only, D only, A and B, A and D, B and D) was added to tanks at 200 cells/ml. Water was continuously circulated through both light and dark chambers by a small pump and water tubing into both light chambers. (c) Treatment tanks were replicated and distributed within a water table with chilled water circulating on the outside of tanks to maintain ambient seawater temperatures. Natural lighting was modulated by a shade cloth

Our study species, *B. asbestinum*, is an abundant octocoral species throughout the Caribbean (Bayer, 1961) and which, following settlement, can simultaneously host as many as six different Symbiodiniaceae species from four deeply divergent genera (Poland et al., 2013). We focused on the early weeks of symbiont uptake in *B. asbestinum*, during which host selection mechanisms appear to be weak, as this provided an opportunity for greater insight into alternative factors, specifically competition among symbionts, in influencing symbiont community structure. Surface brooded *B. asbestinum* larvae were collected from more than 10 adult colonies in a single day in the Middle Florida Keys (24º49′38″N, 80º48′50″W) which appeared to be approximately 2 days old (*B. asbestinum* larvae remain clumped on branches for 3–5 days; Brazeau & Lasker 1990). Larvae were transported to the Keys Marine Laboratory where they were washed several times in 0.45 µm filtered seawater (FSW), and maintained in FSW. Sun‐dried, dead gorgonian branches were provided as a settlement substrate (Coffroth et al., 2006; Poland et al., 2013), onto which larvae attached and metamorphosed into single polyp recruits (Figure 2a).

Branches with attached recruits were distributed among individual 4‐L symbiont treatment tanks. Within these tanks, high‐ and low‐light chambers were created using 1‐L plastic containers; one remained clear and the other was made opaque with black paint covered in tape to prevent chipping. Filtered seawater (0.45 µm) was shared between pairs of high and low light containers and circulated with a small pump. Within each symbiont treatment tank, high‐ and low‐light containers each contained 60–75 settled *B. asbestinum* recruits (Figure 2b). The entire experiment was conducted outdoors under a shade cloth subjected to natural variation in light. Temperature was regulated by circulating chilled freshwater on the outsides of tanks to keep the within‐tank temperature between 27 and 30°C (Figure 2c). Noon light readings over three days were measured with an LI‐193 spherical underwater quantum sensor attached to an LI‐250A meter (Li‐COR Inc., Biosciences) and ranged from 30 to 100 and 200 to 600 µmol photons m^2^ s^−1^ for low‐ and high‐light treatments, respectively. Treatment tanks were randomly rotated within replicate blocks every four days to avoid positional bias with respect to both temperature and light within the larger set up (Figure 2c).

Three symbiont species (*Symbiodinium microadriaticum*,* Brevolium minutum*, and* Durusdinium trenchii*, Family Symbiodiniaceae (previously ITS2‐type A1, B1, and D1a, respectively; LaJeunesse et al., 2018) were sourced from the Buffalo Undersea Reef Research (BURR) Culture Collection (http://burr.bio.buffalo.edu/) for their ability to establish long‐term, successful symbioses with *B. asbestinum* in previous studies (Table 1). The cultures used in the experiments were maintained at the Keys Marine Laboratory (Florida) in f/2 medium (Guillard 1975), at ~27°C, under fluorescent lighting on a 14:10 hr light:dark regime. New batch cultures were regularly restarted to ensure growing and swimming cell populations. Each batch culture was sampled for chloroplast genotyping (Santos et al., 2003) to ensure purity. The use of isoclonal Symbiodiniaceae cultures allowed controlled manipulation of the number of cells of each species made available to the initially aposymbiotic hosts. Species within the ITS2‐type B1 group dominate 1‐ to 2‐year‐old *B. asbestinum* juveniles (Poland et al., 2013); the *B. minutum* culture used in this study has been found in newly settled field recruits, but it is a distinct genotype from that which eventually dominates in juveniles in nature. *S. microadriaticum* and *D. trenchii* are both common constituents of Caribbean cnidarian–dinoflagellate associations (LaJeunesse, Parkinson, & Reimer, 2012; Mellas, McIlroy, Fitt, & Coffroth, 2014; Thornhill, LaJeunesse, Kemp, Fitt, & Schmidt, 2006) and have been found in newly settled *B. asbestinum* in the field (Coffroth et al., 2006; Poland et al., 2013).

**Table 1:** Full description of cultured Symbiodineaceae species

Species	ITS2‐type	cpType^a^	Culture ID
*S. microadriaticum*	A1	A194	04‐503
*B. minutum*	B1	B184	Mf1.05b
*D. trenchii*	D1a	D206	Mf2.2b

a cpType distinguishes phylotypes based on the length heteroplasmy in domain V of chloroplast large subunit (cp23S) ribosomal DNA (Santos et al., 2003).

We used a single, cross‐factorial experimental design nesting light environment within each symbiont treatment, which included either one or two symbiont species. Symbiont treatments included: *S. microadriaticum* only, *B. minutum* only, *D. trenchii* only, *S. microadriaticum* + *B. minutum*, *B. minutum* + *D. trenchii*, and *S. microadriaticum* + *D. trenchii*. Symbionts were added to each tank at a total of 200 cells/ml (100 cells/ml of each symbiont species in mixed inoculations; 760,000 cells total per treatment tank), with three replicate tanks for each treatment. Approximately every four days, the water was fully changed and reinoculated with symbionts throughout the experiment.

The first visible signs of infection were noted at 4–5 weeks after settlement. Whole *B. asbestinum* recruits were sampled for quantitative symbiont genotyping at 6 and 8 weeks postsettlement. At each time point, five randomly selected recruits per symbiont*light treatment were collected from each replicate tank (15 recruits per treatment) by removing the entire *B. asbestinum* recruit and preserving it in 95% EtOH. All polyps were of similar size.

### 2.1 DNA extraction and qPCR

Genomic DNA was extracted from preserved recruits (*n* = 15 for each treatment) using the CTAB protocol for Symbiodiniaceae extraction (Coffroth, Lasker, Diamond, Bruenn, & Bermingham, 1992). Quantitative PCR (qPCR) was used to quantify the abundance of symbiont cells using genus‐specific assays (*Symbiodinium*, prev. Clade A (Winter, 2017); *Breviolum*, prev. Clade B (Cunning, Vaughan, et al., 2015); *Durusdinium*, prev. Clade D (Cunning & Baker, 2013)). All assays were pretested to confirm the applicability of these assays for the three Symbiodiniaceae species employed in this experiment. A set of control recruits (*n* = 5), which were not inoculated with any symbiont cultures, were also tested for the presence of symbionts. DNA extracted from each recruit was assayed with two technical replicates of each genus‐specific primer set. Reaction volumes were 10μL with 5μL Taqman Genotyping Master Mix and 1μL genomic DNA template. Assays were optimized for each target including: *Symbiodinium*, prev. Clade A—150 nM forward primer, 100 nM reverse primer, 150 nM probe; *Breviolum*, prev. Clade B—200 nM forward primer, 300nM of reverse primer, 100 nM probe; *Durusdinium*, prev. Clade D—50 nM forward primer, 75 nM reverse primer, 100 nM probe. All qPCR reactions were performed using a StepOnePlus Real‐Time PCR System (Applied Biosystems) with an initial incubation (2 min @ 50°C, 10 min @ 90°C) followed by 40 cycles of 10s @ 95°C and 1 min @ 60°C. Cycle threshold (*C*_T_) values for each assay were calculated using an automatic baseline interval and relative fluorescence threshold of 0.01.

To convert *C*_T_ values to cell numbers per recruit, standard curves of DNA from known numbers of cells (2,000, 4,000, 16,000, 32,000, and 64,000 cells/sample) of all three cultured Symbiodiniaceae species (extracted using identical methods and volumes as experimental recruits) were amplified in duplicate on each qPCR plate. The known cell numbers for all standard curve amplifications were log‐transformed and modeled as a function of *C*_T_ value and target genus using a linear mixed model with random slopes and intercepts for each qPCR plate. The fitted model was then used to calculate the number of cells of each phylotype in each unknown sample using *C*_T_ value, target genus, and qPCR plate as predictors (all data and analysis code available at https://github.com/shelby26/Mixed-Uptake). We also excluded any cases in which noninoculate symbionts were detected.

### 2.2 Total cell uptake

To examine patterns of symbiont uptake for each of the symbiont species when offered individually, we compared the total number of cells per recruit with a two‐way ANOVA (fixed factors: symbiont treatment, light, and their interaction) at each time point. To conform with the assumptions of ANOVA, cell numbers were first log transformed to fit a normal distribution. When significant differences were detected, a Tukey's HSD post hoc test was performed limited to a priori, within factor comparisons. All analyses were run in R (R Core Team 2015). The value for total cells per recruit within the mixed treatment was incorporated into separate analyses, see below.

### 2.3 Competition in mixed infections

#### 2.3.1 Competitive exclusion

We first tested the ability of each symbiont species to exclude others from entering the symbiosis (i.e., competitive exclusion). Within the mixed inoculation treatments, we pooled data from the two time points and analyzed the frequency with which each symbiont type was present or absent (below the level of detection by qPCR) in *B. asbestinum* recruits. These frequencies were tested in a Pearson's chi‐squared test with Yate's correction for continuity.

#### 2.3.2 Competitive dominance

To test for deviations from the expected values of the de Wit null model in each mixed‐uptake combination, the number of cells of a given symbiont that occurred in the single‐infection recruits was divided by two to generate null model “expected” values for mixed‐infection recruits. While competitive dominance can lead to competitive exclusion over time (which was tested as outlined above), this analysis included data for only those recruits from mixed‐infection treatments in which both intended Symbiodiniaceae strains were detected. For each timepoint, we then fitted a linear mixed model of cell numbers (log‐transformed) as a function of symbiont species, light level, and infection type (single vs. mixed, i.e., expected vs. observed), with random intercepts for each PCR plate. The lsmeans package (Lenth, 2016) was then used to test for significant differences between expected and observed number of cells for each symbiont type within each light level.

## 3 RESULTS

### 3.1 Single‐infection treatments

**Figure 3:**
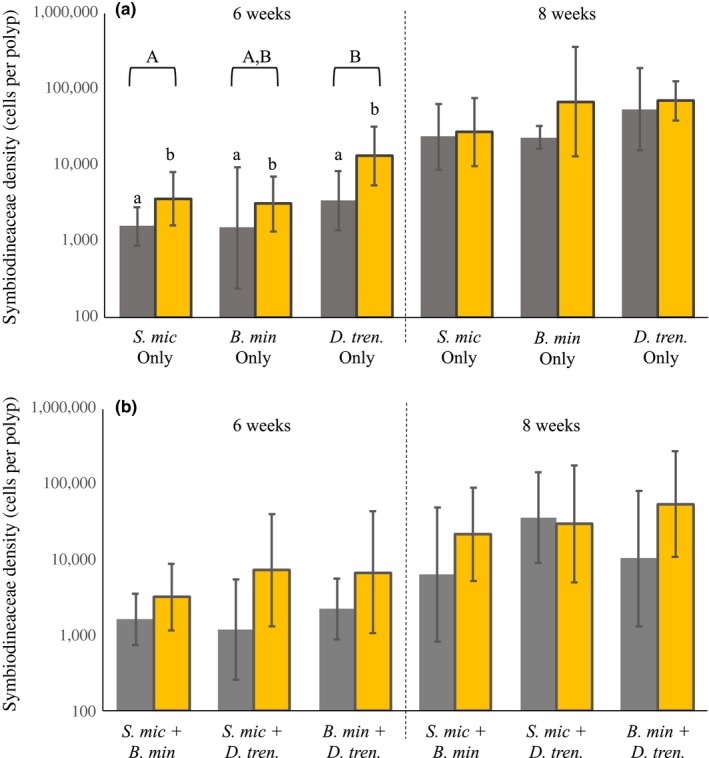
Mean number of symbionts per individual host (*Briareum asbestinum*) recruits in the (a) absence and (b) presence of competition. Low‐light (gray) and high‐light (gold) treatments at 6 weeks (left panel) and at 8 weeks (right panel) following initial inoculation of newly settled recruits. *Symbiodinium microadriaticum* (*S. mic.*), *Breviolum minutum* (*B. min*), *Durusdinium trenchii* (*D. tren.*). Data were log transformed, the geometric mean calculated and back transformed. Error bars represent back transformed 95% confidence intervals. An ANOVA indicated a significant effect of light (small letters) and symbiont (large letters) treatments on symbiont densities within the 6‐week treatment. No significant difference was observed within the 8‐week treatment

All symbiont species were taken up readily when offered individually in both the high‐ and low‐light treatments (Figure 3). At the 6 week time point, polyps harbored 52%–74% fewer symbionts in low‐light treatments relative to high light (*F*_1,50_ = 7.75, *p* = .008). There was also an effect at 6 weeks of symbiont species on cell densities (*F*_2,50_ = 4.12, *p* = .022). A Tukey's HSD post hoc test indicated that the abundance of *D. trenchii* was significantly higher than that of *S. microadriaticum* (*p* = .023), while other comparisons of *S. microadriaticum* versus *B. minutum*, and *B. minutum* versus *D. trenchii* were not significantly different (*p* = .98 and *p* = .18, respectively). There was no interactive effect detected between species and light (*F*_2,50_ = 0.25, *p* = .77). At 8 weeks, there was no significant effect of symbiont type or light environment on the number of symbiont cells per recruit (Symbiont: *F*_2,54_ = 1.74, *p* = .19; Light: *F*_1,54_ = 0.45, *p* = .50; Figure 3). No symbionts were detected in the control recruits. We did not test for an effect of host tissue mass qPCR efficiency; however, we measured similar and increasing symbiont densities through time.

### 3.2 Competition in mixed infections

#### 3.2.1 Competitive exclusion

The detection of only a single symbiont type where two were offered occurred in 26% of recruits sampled from the high‐light treatment and 44% of recruits in the low‐light treatment. Within the low‐light treatment, we found that *B. minutum* was absent (or below the level of detection by qPCR) more frequently than expected when occurring with either *S. microadriaticum* or *D. trenchii* (*χ* ^2^ test; *p* < .05; Table 2). At high light, competitive exclusion was not significant.

**Table 2:** Frequency of competitive exclusions

Treatment	Environment	Symbiont	Present	Absent	Rates *χ* ^2^	*p*
*S. mic. + B. min*	High Light	*S. mic.*	18	6	1.35	.25
		*B. min.*	22	2		
	Low Light	*S. mic.*	20	2	4.36	.04*
		*B. min.*	13	9		
*S. mic. + D. tren.*	High Light	*S. mic.*	24	4	0.89	.35
		*D. tren.*	27	1		
	Low Light	*S. mic.*	23	3	1.11	.29
		*D. tren.*	19	7		
*B. min + D. tren.*	High Light	*B. min.*	16	4	0.20	.66
		*D. tren.*	18	2		
	Low Light	*B. min.*	13	9	6.34	.01*
		*D. tren.*	21	1		

Note

For each mixed symbiont and light treatment, the presence and absence of each symbiont within a sampled *Briareum asbestinum* recruit were recorded. Samples at 6 and 8 weeks were pooled. *Symbiodinium microadriaticum* (*S. mic.*), *Breviolum minutum* (*B. min*), *Durusdinium trenchii* (*D. tren.*). These frequencies were analyzed with a chi‐square test with Yate's correction. Bold values with asterisk indicate that a particular symbiont was excluded significantly more than expected by random chance alone p<0.05.

#### 3.2.2 Competitive dominance

**Figure 4:**
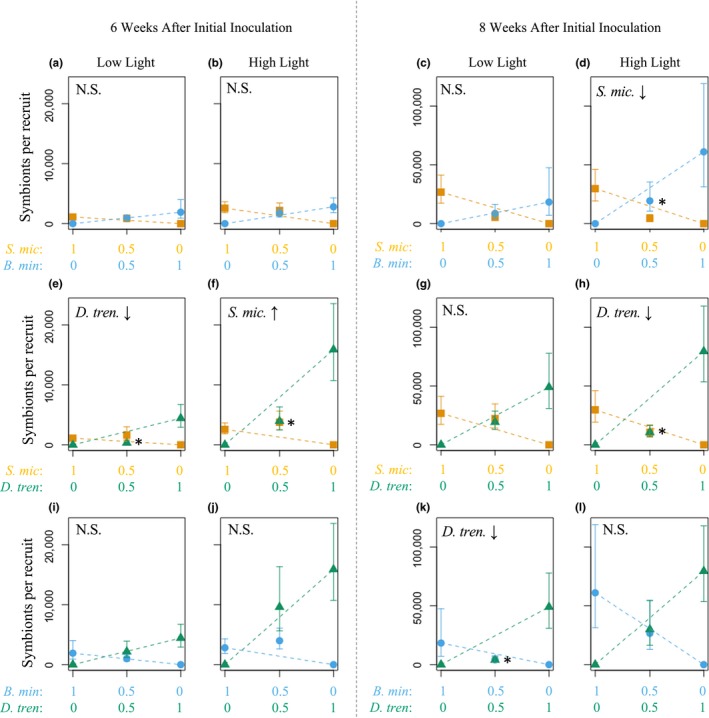
*In‐hospite* symbiont densities (cells per recruit) within single and mixed inoculation treatments. Each symbiont treatment was carried out under both high‐ and low‐light conditions (columns) with data collected at 6 weeks (left panel) and 8 weeks (right panel) following initial inoculation; note different scales on *y*‐axis. *X*‐axis labels show the relative ratio of each symbiont type available for uptake; *Symbiodinium microadriaticum* (*S. mic.*), *Breviolum minutum* (*B. min*), *Durusdinium trenchii* (*D. tren.*). Dashed lines show expectations of the de Wit model based on densities within the single inoculations (see Figure 1). Significant deviations are noted with asterisks, and the letters and arrows in the upper left of the graph indicate direction of deviation within the given symbiont type. Error bars show standard error of the mean

**Figure 5:**
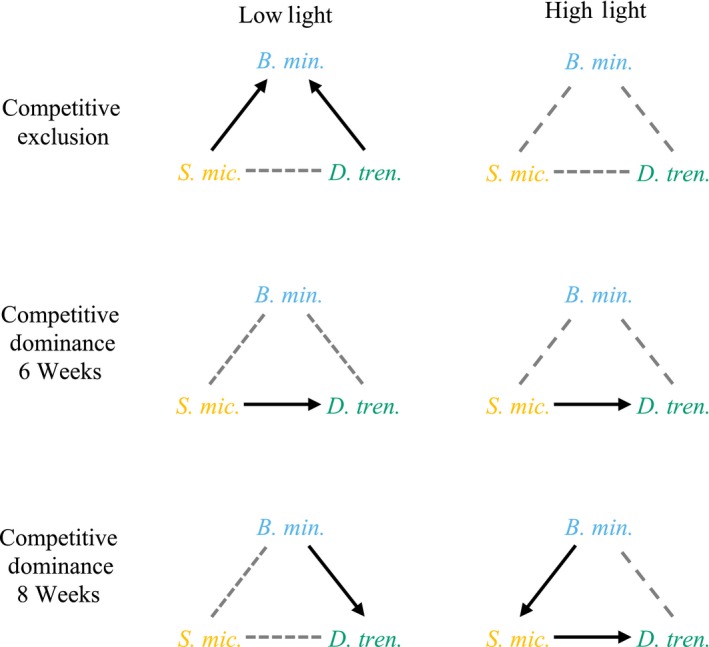
Summary of competitive hierarchy for Symbiodiniaceae species competing for symbiosis with *Briareum asbestinum*. Arrows go from competitive dominants to inferiors based on paired, mixed inoculation treatments under two light environments. Dashed gray connections indicate no significant influence of the presence of one species on the other. Competitive exclusion occurred where one symbiont type completely excluded another (summarized from Table 1), whereas competitive dominance was determined when one species was numerically dominant over the other (detailed from Figure 4). Competitive outcomes were tested at both 6 weeks and 8 weeks following initial inoculations. *Symbiodinium microadriaticum* (*S. mic.*), *Breviolum minutum* (*B. min*), *Durusdinium trenchii* (*D. tren.*)

At 6 weeks postinfection, *D. trenchii* was competitively limited by *S. microadriaticum* under low light (*p* = .011; Figures 4e and 5). A similar pattern was observed in the high‐light treatment, but this difference was not statistically significant (*p* = .174). In the *B. minutum + D. trenchii* treatment, densities of each species were consistent with the null expectation of no competition under both light treatments (*B. minutum* + *D. trenchii* low light: *p* = .928; *B. minutum* + *D. trenchii* high light: *p* = .750; Figures 4i,j and 5). *S. microadriaticum* exceeded densities predicted by the null model in the *S. microadriaticum* + *D. trenchii* high‐light treatment (*p* = .015; Figure 4f), but fell near expected densities within other competition and light treatments (*S. microadriaticum* + *B. minutum* high light: *p* = .299; *S. microadriaticum* + *B. minutum* low light: 0.447; *S. microadriaticum* + *D. trenchii* low light: *p* = .077; Figure 4a,b,i). The number of *B. minutum* cells per polyp was not significantly affected by the presence of either *S. microadriaticum* or *D. trenchii* (*S. microadriaticum* + *B. minutum* high light: *p* = .667; *S. microadriaticum* + *B. minutum* low light: *p* = .928; *B. minutum* + *D. trenchii* high light: *p* = .061; *B. minutum* + *D. trenchii* low light: *p* = .999; Figure 4a,b,i,j).

At 8 weeks postinfection, competitive outcomes were altered. *D. trenchii* was not limited by *S. microadriaticum* at low light (*p* = .659; Figure 4g), but was significantly lower than expected in the presence of *S. microadriaticum* at high light (*p* = .0113; Figure 4h) and in the presence of *B. minutum* at low light (*p* = .005, Figure 4k). However, densities of *S. microadriaticum* were near expectations in the *S. microadriaticum* + *B. minutum* treatment at high light (*p* = .663; Figure 4l). *S. microadriaticum* had lower mean densities than expected in the presence of *B. minutum*, but this was only significant at high light (*S. microadriaticum* + *B. minutum* high light: *p* = .043; *S. microadriaticum* + *B. minutum* low light: *p* = .148; Figure 4c,d). In the presence of *D. trenchii*, *S. microadriaticum* density was not significantly different than expectations (*S. microadriaticum* + *D. trenchii* low light: *p* = .288; *S. microadriaticum* + *D. trenchii* high light: *p* = .490; Figure 4g,h). Densities of *B. minutum* were near the expected densities across all competition and light treatments at 8 weeks (*S. microadriaticum* + *B. minutum* low light: *p* = .959; *S. microadriaticum* + *B. minutum* high light: *p* = .575; *B. minutum* + *D. trenchii* low light: *p* = .404; *B. minutum* + *D. trenchii* high light: *p* = .890; Figure 4c,d,k,l). Results are summarized in Figure 5.

## 4 DISCUSSION

We were able to establish a general competitive hierarchy in which *B. minutum* > *S. microadriaticum* > *D. trenchii*; however, this was offset by competition‐colonization trade‐offs which promote symbiont diversity through time (Figure 5). For example, under light limitation, the rapid acquisition and/or growth of either *S. microadriaticum* or *D. trenchii*, was able to exclude *B. minutum* in some polyps over the first six weeks, but where established, *B. minutum* could transition to dominance. The highly infectious *S. microadriaticum* established symbiosis and proliferated within hosts quickly but was typically competitively inhibited by *B. minutum* where present. The opportunistic *D. trenchii* was ever‐present but often remained at limited abundance in the presence of either competitor.

In isolation, all Symbiodiniaceae species were shown to proliferate more slowly under lower irradiance (Figure 3), supporting a high similarity in requirements among species (at least for light, which is the primary environmental gradient for photosynthetic organisms). In competition, low light frequently led to the exclusion of *B. minutum* from polyps despite simultaneous environmental availability of species pairs (Table 2; Figure 4). While competitive dominance can also lead to exclusion overtime, there was no indication that, where present, *B. minutum* densities were declining or lower than expected (Figure 4: competitive dominance) suggesting that these species had yet to establish with the polyps. Instead, priority effects, in which the earliest arrivals exclude later arriving symbiont types (Fukami, 2015; Kennedy, Peay, & Bruns, 2009; Werner & Kiers, 2015), can influence symbiont community structure, particularly among species with high niche overlap (e.g., light availability within the host). Such cases of habitat preemption may be initiated with competitive behaviors outside of the host. Swarming, that is maintaining high densities near available hosts, has been reported for both *S. microadriaticum and D. trenchii* (Yamashita, Suzuki, Kai, Hayashibara, & Koike, 2014). Subsequent priority effects then occur where early arriving species have a large impact on that niche (Palmer et al., 2002), and where late arrivals are highly sensitive to niche availability (Fukami, Beaumont, Zhang, & Rainey, 2007). Indeed, the cell size of both *S. microadriaticum* and *D. trenchii* is large relative to *B. minutum* (Biquand et al., 2017; LaJeunesse, 2001; LaJeunesse, Lambert, Andersen, Coffroth, & Galbraith, 2005; Suggett, Goyen, & Evenhuis, 2015) which could more rapidly lead to light limitation via shading (Cunning & Baker, 2013) as these larger cells populate host tissues. Furthermore, larger Symbiodiniaceae cells tend to have an increased ability for light‐harvesting which may lead to a positive feedback in their competitive advantage over smaller cells (Suggett et al., 2015).

The long‐term trajectory of *B. asbestinum* juveniles in favor of *Breviolum* spp. has been demonstrated in both the laboratory and field (Poland & Coffroth, 2017, 2019; Poland et al., 2013). Indeed, numerous coral species establish predictable symbiont associations over time, regardless of the initial composition of symbiont communities (Abrego et al., 2009; Little et al., 2004; McIlroy & Coffroth, 2017; Poland & Coffroth, 2017; Poland et al., 2013; Quigley, Willis, & Bay, 2016). However, even short‐term competition and succession among symbionts may have important consequences for host fitness. While we did not quantify the impact of symbiont composition on hosts in this study, a laboratory study by (Poland & Coffroth, 2019) found that, by 3 months of age (1 month beyond our own study), *B. asbestinum* hosting *Symbiodinium* sp. or mixed communities of *Symbiodinium* sp. and *Breviolum* spp. had slower growth (i.e., polyp budding rates) and higher mortality relative to those hosting only *Breviolum*. In the field, selection for fast succession to optimal symbionts may be even more pronounced, particularly for juveniles corals that have an inverse relationship between size and mortality (Edmunds & Gates, 2004). In fact, this seems to be the case with *B. asbestinum* where the majority of field‐reared polyps are dominated by *Breviolum* by three months (Poland et al., 2013). In symbioses, infectivity and high rates of *in‐hospite* proliferation are generally associated with parasitism because of their demand on a shared pool of nutritional resources (Baker et al., 2018; Sachs & Wilcox, 2006). However, balancing selection for both competitive (i.e., self‐promoting) and mutualistic (i.e., promoting host growth and fitness) traits may ultimately lead to evolution of predictable and beneficial host‐symbiont associations even in the absence of host control.

Despite the global ubiquity of members of *Durusdinium* at very low relative abundance in corals (Silverstein, Correa, & Baker, 2012; Tong et al., 2017), the poor competitive ability of *D. trenchii* shown here may contribute to its uncommonness as a dominant symbiont, except following bleaching (the stress‐induced loss of symbionts from the host). Furthermore, the fact that *D. trenchii* is not particularly competitive under the conditions studied here may promote the reversion to alternative dominant symbiont types following recovery (Jones et al., 2008; Thornhill et al., 2006) assuming conditions return to what they were prior to bleaching. While not studied in *B. asbestinum*, members of the genus *Durusdinium* are known for aiding recovery following bleaching and increasing resistance to thermal stress, but provide lesser nutritional benefits to coral hosts compared with other symbiont types under nonstressful conditions (Baker, Andras, Jordán‐Garza, & Fogel, 2013; Cantin, Oppen, Willis, Mieog, & Negri, 2009; Little et al., 2004). However, these relative benefits may also change depending on environmental condition (Cunning, Gillette, Capo, Galvez, & Baker, 2014). In this way, competitive outcomes can also underpin the complementary or even synergistic benefits of multispecies mutualisms on lifetime coral fitness (Palmer et al., 2010).

We were able to demonstrate that competition among symbionts influences the colonization of new hosts, but we found that our competitive hierarchies were not generalizable through time with variable outcomes at 6 and 8 weeks. One explanation is that a host is not a static habitat. Changes in the host environment, distribution of symbiont cells among tissues, and/or nutrient sharing can occur as hosts grow (Lecointe, Domart‐Coulon, Paris, & Meibom, 2016) which may alter the competitive hierarchy. Furthermore, while the small size of recruits limited our ability to assess both symbiont genetics and host tissue mass simultaneously, differences in recruit growth among treatments may have fed‐back into competitive outcomes. Symbionts also become more densely packed into host tissues overtime. The approximately 10‐fold increase in symbiont densities between 6 and 8 weeks across light treatments may allow for density‐dependent effects on competitive abilities (Cunning, Vaughan, et al., 2015). Thus, long‐term associations may favor those symbionts that compete well and establish in later host ontogenetic stages. Lastly, research focused on host control of *in‐hospite* symbiont communities provides some foundation for mechanisms of symbiont recognition and regulation which coincide with coral ontogeny and the development of immune responses, which are limited until at least three months (Coffroth et al., 2001; McIlroy & Coffroth, 2017; Nozawa & Loya, 2005; Poland & Coffroth, 2017; Poland et al., 2013; Puill‐Stephan, Willis, Abrego, Raina, & Oppen, 2012). Host control may act in concert with or to override mechanisms of symbiont competition in order to avoid associations with nonoptimal or parasitic symbionts and to promote the evolutionary stability of coral–algal mutualisms.

Previously, models that compared hosts with single versus mixed symbiont assemblages have been used to understand coevolution (Gomulkiewicz, Nuismer, & Thompson, 2003; Hoeksema & Kummel, 2003), to better predict the effect of mutualists on host–enemy interactions (McKeon, Stier, McIlroy, & Bolker, 2012; Morris et al., 2007; Palmer et al., 2008), to predict spatial and environmental characters that promote mutualism function (Boza & Scheuring, 2004; Doebeli & Knowlton, 1998) and to understand host ontogeny (Palmer et al., 2010). The compact, easily replicated, coral–algal mutualism presents an excellent model system to further explore these phenomena and provide new perspectives on the consequences of diversity and flexibility in symbiosis in general. Future research, aided by techniques that can quantify absolute and relative abundances of specific symbionts in mixed associations (e.g., qPCR, high‐throughput sequencing, and genetic tagging) and more directly demonstrate their effects on host fitness (e.g., physiology) will continue to reveal the ecology and evolution of diverse symbioses ubiquitous throughout the earth's ecosystems.

## CONFLICT OF INTEREST

All authors declare that there are no competing financial interests in relation to the work described.

## AUTHOR CONTRIBUTIONS

SEM and MAC conceived the projects and executed experiments, RC and ACB developed genetic methodologies, SEM and RC ran the genetic assays and data analyses, all authors contributed to the writing of the manuscript and have approved the final version.

## Data Availability

Upon publication, all data and analysis code will be available at https://github.com/shelby26/Mixed-Uptake.
